# The ruthenium compound KP1339 potentiates the anticancer activity of sorafenib *in vitro* and *in vivo*^☆^

**DOI:** 10.1016/j.ejca.2013.05.018

**Published:** 2013-10

**Authors:** Petra Heffeter, Bihter Atil, Kushtrim Kryeziu, Diana Groza, Gunda Koellensperger, Wilfried Körner, Ute Jungwirth, Thomas Mohr, Bernhard K. Keppler, Walter Berger

**Affiliations:** a Institute of Cancer Research, Department of Medicine I, Medical University Vienna, Austria; b Comprehensive Cancer Center of the Medical University Vienna, Austria; c Research and Platform “Translational Cancer Therapy Research” Vienna, Austria; d Department of Chemistry, Division of Analytical Chemistry, University of Natural Resources and Applied Life Sciences-BOKU, Austria; e Department of Environmental Geosciences, University of Vienna, Vienna, Austria; f University of Vienna, Institute of Inorganic Chemistry, Vienna, Austria

**Keywords:** Sorafenib, Ruthenium, Drug accumulation, P38, STAT3, Cell cycle arrest, Synergism

## Abstract

KP1339 is a promising ruthenium-based anticancer compound in early clinical development. This study aimed to test the effects of KP1339 on the *in vitro* and *in vivo* activity of the multi-kinase inhibitor sorafenib, the current standard first-line therapy for advanced hepatoma. Anticancer activity of the parental compounds as compared to the drug combination was tested against a panel of cancer cell lines with a focus on hepatoma. Combination of KP1339 with sorafenib induced in the majority of all cases distinctly synergistic effects, comprising both sorafenib-resistant as well as sorafenib-responsive cell models. Several mechanisms were found to underlie these multifaceted synergistic activities. Firstly, co-exposure induced significantly enhanced accumulation levels of both drugs resulting in enhanced apoptosis induction. Secondly, sorafenib blocked KP1339-mediated activation of P38 signalling representing a protective response against the ruthenium drug. In addition, sorafenib treatment also abrogated KP1339-induced G2/M arrest but resulted in check point-independent DNA-synthesis block and a complete loss of the mitotic cell populations. The activity of the KP1339/sorafenib combination was evaluated in the Hep3B hepatoma xenograft. KP1339 monotherapy led to a 2.4-fold increase in life span and, thus, was superior to sorafenib, which induced a 1.9-fold prolonged survival. The combined therapy further enhanced the mean survival by 3.9-fold. Synergistic activity was also observed in the VM-1 melanoma xenograft harbouring an activating braf mutation. Together, our data indicate that the combination of KP1339 with sorafenib displays promising activity *in vitro* and *in vivo* especially against human hepatoma models.

## 1 Introduction

Therapy with cytotoxic compounds or small molecule inhibitors is a major strategy to treat human cancer at the disseminated stage. Based on improved technological possibilities during the last decades, the identification of tumour-specific alterations resulted in novel targeted treatment strategies with improved effectiveness. However, even for new targeted therapeutics, the occurrence of drug resistance and unwanted side-effects remains a major obstacle for successful long-term treatment.^1,2^ Thus, in addition to the development of new drugs, especially the enhancement of activity by combination of drugs is in the focus of clinical research.^1^ However, besides cis- and oxaliplatin, metal drugs have been only rarely included in such investigations. Recently, the investigative anticancer ruthenium compound indazolium *trans*-[tetrachlorobis(1H-indazole)ruthenate(III)] (KP1019) demonstrated promising anticancer activity in a clinical phase I study with frequent disease stabilisation.^3,4^ Notably, only mild treatment-related toxicities were observed, encouraging further clinical development of KP1019.^4^ For better clinical application, the water-soluble sodium salt of KP1019, termed KP1339, has been recently developed and has recently successfully finished a clinical phase I trial where profound activity especially against neuroendocrine tumours (NET) were observed.^5^

Sorafenib (BAY 43-9006, Nexavar) is a multi-targeted tyrosine kinase inhibitor that exerts potent anti-angiogenic and antitumour activities. Sorafenib is approved for the treatment of advanced renal cell carcinoma as well as unresectable hepatocellular carcinoma and is currently developed for other solid tumours.^2,6^ Initially, this small molecule inhibitor was identified as an inhibitor of Raf serine/threonine kinases. Additionally, it inhibits several oncogenic and/or pro-angiogenic receptor tyrosine kinases, such as FMS-like tyrosine kinase 3 (Flt3), the mast/stem cell growth factor receptor (c-kit), the RET proto-oncogen, vascular endothelial growth factor receptor (VEGFR) 1/2/3, and the platelet-derived growth factor receptor (PDGFR) 1. However, also some non-kinase targets for sorafenib have been suggested.^6–8^ Recent studies demonstrated interference of sorafenib with several cellular transport mechanisms involved in drug resistance such as several ATP-binding cassette (ABC) transporters or the non-ABC transporter RLIP76.^9,10^ However, the impact of these effects on the activity and pharmacological characteristics of other drugs remains widely unexplored. This study aimed to evaluate the activity of the combination between KP1339 and sorafenib against solid human cancer cell models *in vitro* and *in vivo*.

## 2 Material and methods

### 2.1 Chemicals

KP1339 was synthesised as published.^11^ Kinase inhibitors were purchased from LC laboratories (Woburn, United States of America [USA]). For *in vitro* studies, the compounds were dissolved in dimethyl sulfoxide (DMSO) (end-concentrations always below 1%). All other substances were from Sigma–Aldrich (St. Louis, USA).

### 2.2 Cell lines

The human cancer cell lines, their source as well as the used cell culture media supplemented with 10% fetal calf serum (PAA, Linz, Austria) are given in Supplementary Table 1. Cultures were periodically checked for *Mycoplasma* contamination.

### 2.3 Cytotoxicity assays

Cell viability was determined by 3-(4,5-dimethylthiazol-2-yl)-2,5-diphenyltetrazolium (MTT) assay as published.^11,12^ Cytotoxicity was calculated using the Graph Pad Prism software (using a point-to-point function) and was expressed as IC_50_ values calculated from full dose–response curves (drug concentrations inducing a 50% reduction of cell number in comparison to untreated control cells cultured in parallel). Synergism is expressed by the combination index (CI) according to Chou and Talalay^13^ using CalcuSyn software (Biosoft, Ferguson, MO, USA). CI < 0.9, CI = 0.9–1.2 or CI >1.2 represent synergism, additive effects and antagonism, respectively.

### 2.4 Cell cycle analyses

Cell cycle distribution of drug-treated cells was determined by propidium iodide staining and flow cytometry as published.^12^

### 2.5 Total ruthenium (Ru) uptake levels

Intracellular Ru levels were determined as recently described.^11^ A detailed description is included in Supplementary material and methods.

### 2.6 Western blot

Total proteins were isolated and Western blots performed as described.^12^ Primary antibodies used are given in Supplementary Table 2. Additionally, secondary horseradish peroxidase-labelled antibodies (Santa Cruz Biotechnology) were used at dilutions of 1:10000.

### 2.7 Animals

Eight-week-old female CB-17 scid/scid (SCID) mice were purchased from Harlan Laboratories (San Pietro al Natisone, Italy). The animals were kept in a pathogen-free environment and every procedure was done in a laminar airflow cabinet. Experiments were carried out according to the Austrian and the Federation of Laboratory Animal Science Associations (FELASA) guidelines for animal care and protection.

### 2.8 Xenograft experiments

Hep3B (10^6^ with 10% matrigel) or VM-1 cells (1.5 × 10^6^) were injected subcutaneously into the right flank. When tumour nodules reached a mean size of 25 mm^3^, animals were treated with sorafenib orally (in Cremophor EL, diluted in ethanol and then 1:10 with deionised water), KP1339 intravenously (in citrate buffer pH 3.5) or both. Tumour size was assessed by caliper measurement. Tumour volume was calculated using the formula: (length × width^2^)/2. The combined drug effects were assessed as tumour delay (Td) defined as the time in days to reach a tumour volume of 300 mm^2^.

### 2.9 Quantification of sorafenib

A new liquide chromatography- mass spectroscopy (LC-MS) method has been established to detect the intracellular accumulation of sorafenib. A detailed description is included in Supplementary material and methods.

## 3 Results

### 3.1 Synergistic activity of KP1339 with sorafenib against diverse malignant tumour types

**Fig. 1:**
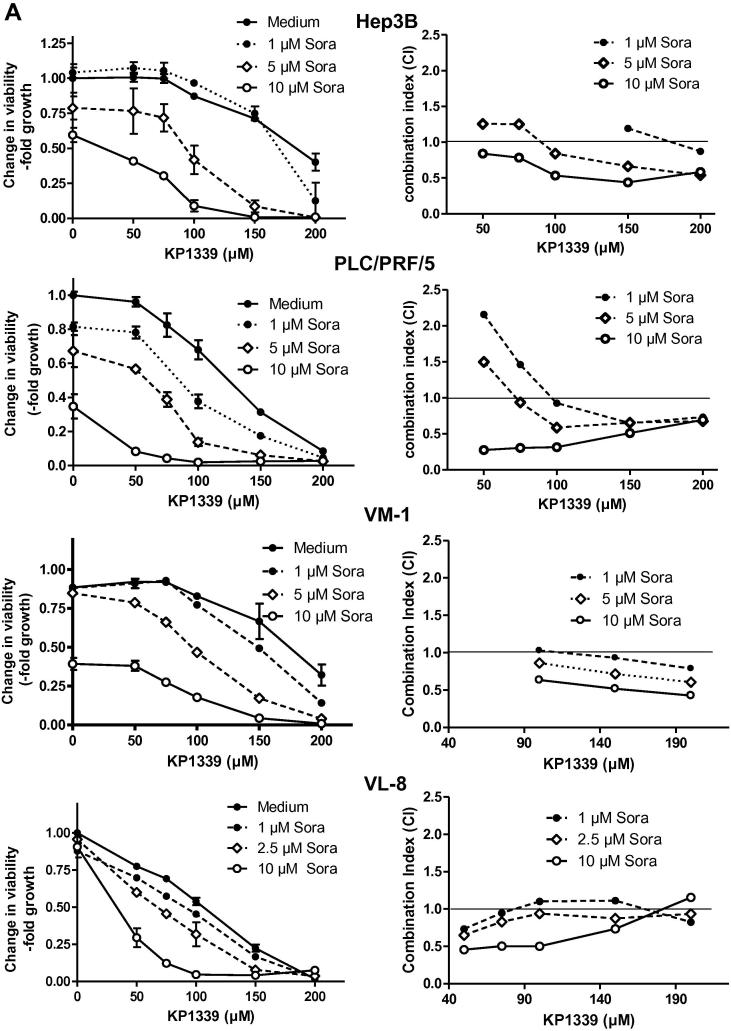
Anticancer activity of KP1339 in combination with sorafenib. The indicated cell lines were treated for 72 h with KP1339 and sorafenib. Left panel: Viability was evaluated by MTT assay. Values given are means ± standard deviations of one representative experiment performed in triplicates. Right panel: combination index (CI) was calculated using CalcuSyn software (compare Section 2).

The effects of sorafenib on the anticancer activity of KP1339 were tested in malignant cell lines of diverse origin with a focus on hepatoma (*n* = 12). Results obtained are shown in Fig. 1 and in Supplementary Figs. S1–S3. Most cell lines exhibited responsiveness to sorafenib mono-treatment with a mean IC_50_ value below 10 μM (Table 1). Only, in case of VL-8, Hep3B, HCC1.1 and SW480 IC_50_ values >10 μM were observed. The IC_50_ values for KP1339 mono-therapy were between 45 and 200 μM (Table 1). For the drug combination, sorafenib co-treatment with KP1339 showed additive to strong synergistic effects in all cell lines investigated. Notably, the combination of KP1339 with 10 μM sorafenib was highly synergistic with CI values between 0.1 and 0.8 and especially KP1339 concentrations between 100 and 200 μM synergistically increased the anticancer activity of sorafenib. Notably, the KP1339 and sorafenib drug combination exhibited synergistic activity in sorafenib-resistant (HCC1.1, Hep3B, VL-8 and VM-1) and sorafenib-responsive cell lines (PLC/PRL/5, HepG2 and HCC2). This distinct synergism was in contrast to several other kinase inhibitors. Thus, weak synergism was also observed with (the VEGFR-1/2/3, PDGFR-ß and c-kit inhibitor) sunitinib but not for axitinib (VEGFR1/2/3, PDGFRα/ß, c-kit) or vemurafenib (targeting braf V600E). Distinct antagonism was observed with (the abl, PDGFR3 and c-kit inhibitor) gleevec (Supplementary Fig. 4).

### 3.2 Effects of sorafenib on KP1339-induced apoptosis

**Fig. 2:**
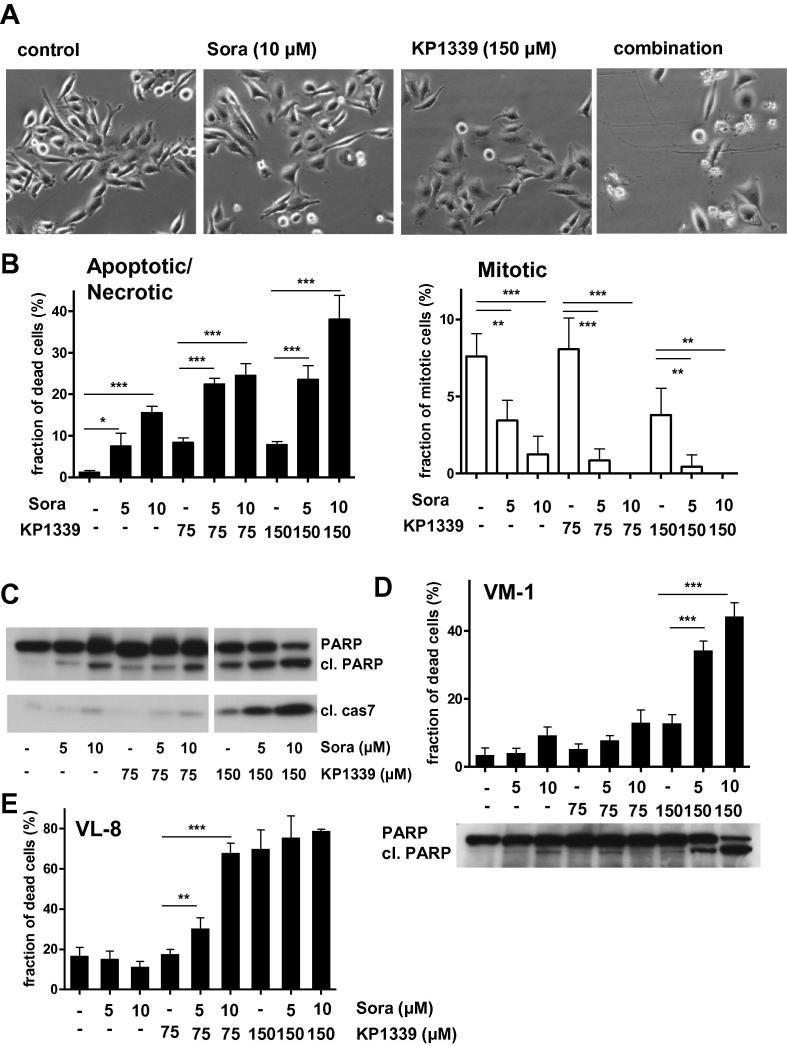
Impact of sorafenib on apoptosis-induction by KP1339 treatment. (A) Morphologic changes in Hep3B cells after 24 h drug treatment. Photomicrographs shown were taken with a 10x objective and phase contrast settings using Nikon Eclipse TE300 (Nikon Instruments, Japan). (B) Induction of apoptosis was determined in Hep3B cells after 24 h treatment. Nuclei of methanol/aceton-fixed cells were stained by DAPI and morphological features of 300–500 nuclei of at least two slides for each concentration were analysed. Percentages of normal and apoptotic/necrotic cells are shown on the left panel, while percentages of mitotic cells are shown on the right panel. For statistical analyses 2-way Anova with Bonferroni post correction was performed (^∗∗∗^*p *< 0.001, ^∗∗^*p *< 0.01, ^∗^*p *< 0.05). (C) Cleavage of poly(ADP-ribosyl)polymerase (PARP) and caspase-7 in Hep3B cells after 24 h drug treatment. (D) Induction of apoptosis and PARP cleavage was determined in VM-1 cells after 24 h treatment. (E) Induction of apoptosis in VL-8 after 24 h treatment. For statistical analyses 2-way Anova with Bonferroni post correction was performed (^∗∗∗^*p *< 0.001, ^∗∗^*p *< 0.01, ^∗^*p *< 0.05).

Morphological changes induced in Hep3B cells by the mono-treatments or drug combination are shown in Fig. 2A. The cells were analysed by 4́,6-diamidino-2-phenylindole (DAPI) staining to evaluate chromatin condensation as a parameter for programmed cell death induction (Fig. 2B). In accordance to already published observations, sorafenib^14^ as well as KP1339^11^ mono-treatment induced apoptosis in a dose-dependent manner. Combination of KP1339 with sorafenib synergistically increased the number of apoptotic cells. This was accompanied by enhanced poly(ADP-ribosyl)polymerase (PARP) and caspase 7 cleavage (Fig. 2C). Similar results were obtained using the braf-mutated melanoma model VM-1 and the NSCLC-model VL-8 (Fig. 2D and E). In addition to the enhanced cell-death inducing potential, a distinct loss of the mitotic cell population was found in all combination-treated samples (Fig. 2B, right panel).

### 3.3 Effects of the KP1339/sorafenib combination on cell cycle progression

**Fig. 3:**
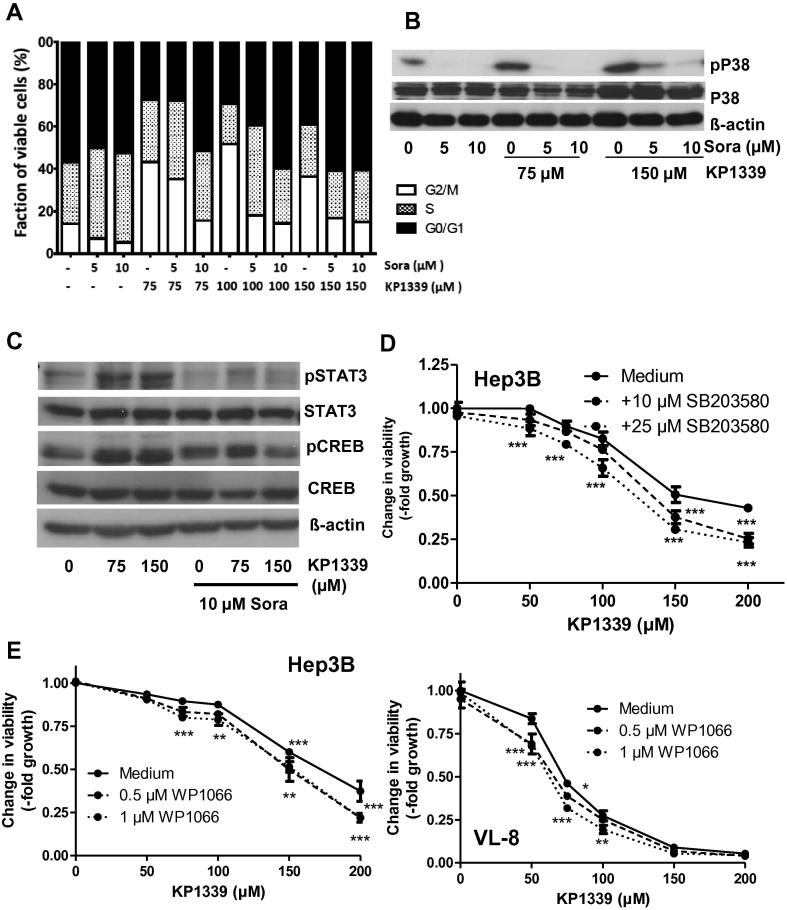
Impact of sorafenib on KP1339-induced cell cycle arrest and P38 signalling in Hep3B cells. (A) Cell cycle distribution was analysed by PI-staining after 24 h treatment. Percentages of 25000 cells in G0/G1, S and G2/M phase of cell cycle were calculated by Cell Quest Software. (B) KP1339-induced changes in P38 phosphorylation in combination with sorafenib were determined after 24 h treatment by Western blotting. (C) The effect of sorafenib co-treatment on KP1339-induced changes in phosphorylation status of P38 downstream signalling proteins was investigated after 24 h treatment by Western blotting. The impact of P38 inhibition by SB203580 (D) (after 30 min preincubation) or STAT3 inhibition by WP1066 (E) on the anticancer activity of KP1339 was tested in the indicated cell lines by MTT assay after 72 h treatment. Values given are relative means and standard deviation (SD) from at least 2 independent experiments performed in triplicates. For statistical analyses 2-way Anova with Bonferroni post correction was performed (^∗∗∗^*p *< 0.001, ^∗∗^*p *< 0.01, ^∗^*p *< 0.05).

Next, the effects of the KP1339-sorafenib combination on cell cycle distribution were analysed. In several investigated cell lines (Figs. 3A and Supplementary Fig. S5), KP1339 treatment resulted in a distinct increase of cells in G2/M phase. Sorafenib mono-treatment also effected cell cycle distribution leading to an increase of cells either in G0/G1 or S phase. In the combination settings sorafenib strongly suppressed the KP1339-induced increase of cells in G2/M at all concentrations tested. We have previously reported that treatment with KP1019 induces oxidative stress,^15^ which is known to activate the P38 mitogen-activated protein (MAP) kinase pathway^16^ and in turn G2/M cell cycle arrest.^17–19^ Consequently, the effect of sorafenib on KP1339-mediated P38 activation was analysed. As shown in Fig. 3B and Supplementary Fig. S6, KP1339 treatment distinctly increased phosphorylation of P38, which was inhibited by sorafenib cotreatment. A similar effect was also seen for the phosphorylation of the P38 downstream signalling proteins signal induce and activator of transcription 3 (STAT3) and cAMP response element-binding protein (CREB) (Fig. 3C). Consequently, it was also tested whether the P38 inhibitor SD203580 or the STAT3 inhibitor WP1066 synergise with KP1339. Indeed, inhibition of P38 as well as its downstream target STAT3 slightly increased the anticancer activity of KP1339 (Fig. 3D and E).

### 3.4 Effects of sorafenib cotreatment on intracellular Ru accumulation and vice versa

**Fig. 4:**
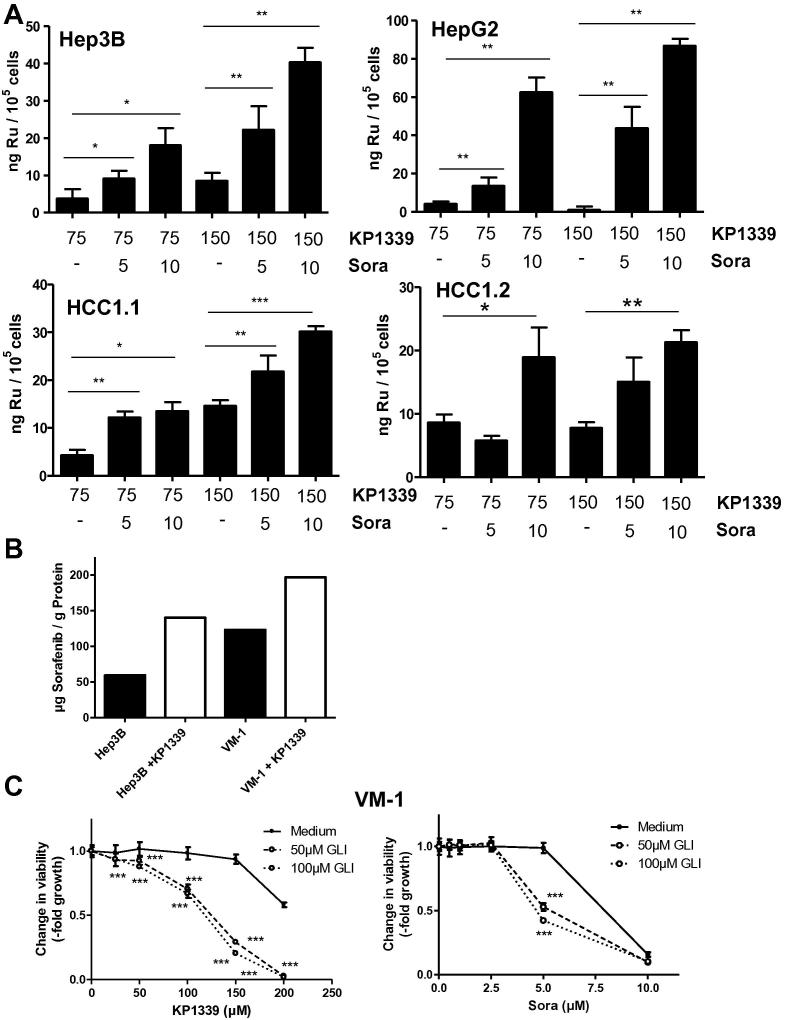
Effects of the combination on the intracellular drug accumulation. (A) Effect of sorafenib cotreatment on the total ruthenium (Ru) levels in the indicated cell lines was determined after 3 h KP1339 exposure by inductively-coupled plasma mass spectroscopy (ICP-MS). Values given are relative means and standard deviation (SD) from at least two independent experiments performed in triplicates. (B) Effect of KP1339 cotreatment on cytosolic sorafenib levels was determined by LC-MS measurements as described in Section 2. (C) VM-1 cells were treated with KP1339 or sorafenib together with the indicated concentrations of GLI for 72 h. Viability was evaluated by MTT assay. Values given are means ± standard deviations of one representative experiment performed in triplicates (^∗∗∗^*p *< 0.001, ^∗∗^*p *< 0.01, ^∗^*p *> 0.05).

Intracellular ruthenium levels were determined to investigate whether sorafenib increases cellular KP1339 uptake. Sorafenib co-treatment significantly enhanced total KP1339 uptake in all tested cell lines (Fig. 4A). With respect to the sorafenib accumulation, the combination of 75 μM KP1339 with 10 μM sorafenib induced a 2.4-fold increase of the tyrosine kinase inhibitor in Hep3B and a 1.6-fold increase in VM-1 cells (Fig. 4B). These effects were not based on general changes in cellular membrane integrity (Supplementary Figs. S7 and S8) Notably, also combination experiments using the panABC transporter inhibitor glibenclamide (GLI) resulted in synergistic activity with both sorafenib as well as KP1339 (Fig. 4C and Supplementary Fig. S9). This indicates that competitive ABC transporter inhibition might be involved in the synergism of KP1339 with sorafenib.

### 3.5 Synergistic activity of KP1339 with sorafenib *in vivo*

**Fig. 5:**
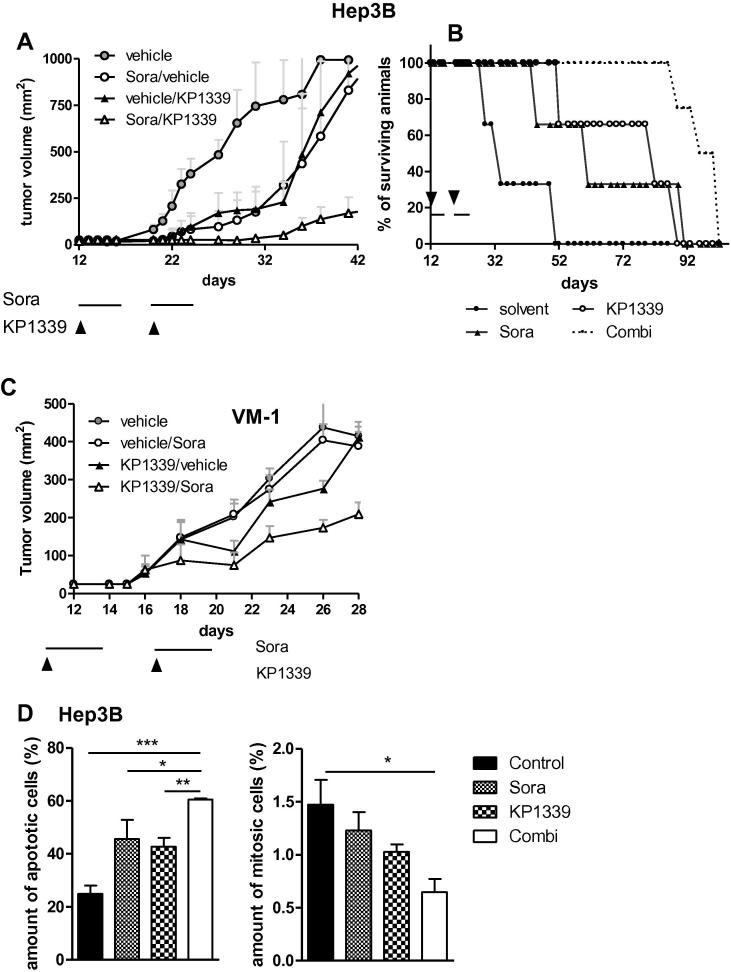
Anticancer activity of the KP1339/sorafenib combination *in vivo*. Hep3B xenografts were grown in Balb/c SCID mice and treated with KP1339 (30 mg/kg; intravenous (i.v.); once a week) and/or sorafenib (25 mg/kg; p.o.; five consecutive days per week) for 2 weeks. (A) effect of treatment on tumour growth; (B) effect of treatment on overall survival. (C) VM-1 xenografts were grown in Balb/c SCID mice and treated with KP1339 (30 mg/kg; i.v.; once a week) and/or sorafenib (25 mg/kg; p.o.; five consecutive days per week) for 2 weeks. (D and E) Hep3B xenografts were grown in Balb/c SCID mice and treated with the above described drug dose as indicated at the figure axis. At the last day of treatment tumour samples were collected, paraffin-embedded and slices prepared. Percentages of apoptotic (D) and mitotic (E) cells in H/E-stained tumour sections (*n* = 3 from *n* = 4 mice) were evaluated microscopically by counting. For statistical analyses 2-way Anova with Bonferroni post correction was performed (^∗^*p *< 0.05, ^∗∗^*p *< 0.01, ^∗∗∗^*p *< 0.001) using Graph Pad Prism software.

The influence of KP1339 on the anti-hepatoma activity of sorafenib *in vivo* was tested using a Hep3B SCID mouse xeno-transplantation model. All treatment schemes were well tolerated and the mice did not exhibit any symptoms of toxicity, such as fatigue, or significant weight loss (Supplementary Fig. 10A). With regard to the anticancer activity, KP1339 as well as sorafenib monotreatment induced a distinct delay in tumour growth (Fig. 5A). Thus, Td values (day when tumour volumes reached 300 mm^3^) increased from 22.8 days to 34.7 and 33.7 days, respectively. In the drug combination, a strong anti-tumour effect was observed which led to a Td value of 47 days. This pronounced anticancer activity resulted in life prolongation in all treated groups (Fig. 5B): KP1339 treatment induced a 2.4-fold increase in life span with a mean survival of 80 days as compared to 33 days in the control group. Sorafenib mono-treatment induced a 1.9-fold increase in survival (60 days). Most importantly, the combined treatment with KP1339 and sorafenib was most effective with a significant increase in mean survival by 3.9-fold to 96 days. Comparably, synergistic activity of KP1339 with sorafenib was observed in braf V600E-positive VM-1 cells (Fig. 5C). To evaluate whether the pronounced anticancer activity of the combination is based on enhanced apoptosis induction, tumour samples of Hep3B-bearing animals were collected after short time treatment (Supplementary Fig. 10B) and analysed by hematoxylin/eosin (H/E) staining. As shown in Fig. 5B, treatment with the drug combination resulted in significantly increased apoptosis levels as well as decrease in cells displaying mitotic features.

## 4 Discussion

The multi-kinase inhibitor sorafenib is the current standard therapeutic for advanced hepatoma. However, tumour progression based on resistance development is frequently observed after a few months of monotherapy^2^ implicating the need for combination strategies. In this study, we demonstrated that the novel ruthenium compound KP1339 distinctly enhances the anticancer activity of sorafenib *in vitro* and *in vivo*. Additionally, several molecular mechanisms underlying this synergism were identified. Sorafenib mediates attenuation of KP1339-induced G2/M arrest and inhibits activation of the protective P38 pathway. Additionally, increased intracellular accumulation of both drugs was observed in the combination setting. Together this resulted in enhanced apoptosis induction and prolonged disease control *in vivo*.

KP1339 is a novel ruthenium compound currently tested with promising results in a clinical I/II trial.^5^ KP1339 treatment is known to generate intracellular reactive oxygen species (ROS) probably caused by the reduction-induced redox cycling^15,20^ and to induce apoptosis via the mitochondrial pathway.^11,21^ Here, we demonstrated that KP1339 in addition to apoptosis induction potently arrests cell cycle progression in G2/M phase which is accompanied by activation of P38 signalling. P38 is a stress kinase, which has (comparable to other MAP kinsases such as ERK) multiple downstream targets and, consequently, is also involved in the regulation of other stress-induced effects such as apoptosis induction.^16,22^ There is increasing evidence that activation of P38 MAPK by stress stimuli may not necessarily promote cell death but instead also enhance cell survival through activation of a transient G2/M cell cycle checkpoint and successful DNA damage repair.^18,19^ This corroborates with our results that treatment with sorafenib blocks KP1339-induced activation of the P38 signalling pathway. However, as specific P38 inhibition only modestly (but significantly) enhanced the anticancer activity of KP1339, it was concluded that overcoming the KP1339-induced G2/M arrest might not be the only mechanism underlying the observed synergistic activity of KP1339 with sorafenib. This is further supported by the observation of (although distinctly weaker) synergistic activity of KP1339 with the tyrosine kinase inhibitor sunitinib, which shares several targets (VEGF, PDGF, KIT, FLT3 and RET) with sorafenib but does not inhibit P38 phosphorylation.^23^ Consequently, additional mechanisms underlying the synergism of KP1339 with sorafenib must exist.

Sorafenib as well as sunitinib have been recently reported to inhibit several ATP-binding cassette (ABC) transporters,^10,24^ which are responsible for multidrug resistance due to enhanced efflux of diverse drugs including doxorubicin, etoposide, taxol or imatinib.^25^ In addition, both drugs are substrates for the non-ABC transporter RLIP76 and were consequently suggested to competitively interfere with the export of glutathione conjugates.^9,10^ Thus, the impact of sorafenib on KP1339 drug accumulation was considered as one possible mechanism underlying the observed synergism. Indeed, sorafenib cotreatment distinctly increased intracellular KP1339 levels, and KP1339 conversely enhanced sorafenib accumulation. Moreover, the panABC transporter inhibitor glibenclamide potently increased the activity of sorafenib as well as KP1339. These data indicate that KP1339 and sorafenib share a common export mechanism. Notably, we have reported before that the activity of KP1019 (the precursor of KP1339) is not limited by ABCG2, ABCC1 and ABCC2 but the metal drug is a weak substrate for ABCB1.^26^ However, the synergistic activity of KP1339 with sorafenib does not correlate with intrinsic ABCB1 expression and no enhanced synergism was observed in ABCB1- or ABCC1-overexpressing cell models as compared to the parental cell lines (data not shown). Nevertheless, cancer cells (especially hepatoma cells) express multiple ABC transporters.^27–29^ Thus, the involvement of other ABC transporters in the synergism of KP1339 with sorafenib seems likely. Moreover, non-ABC transporter systems such as RLIP76 also need to be considered^30^ especially as additional functions in glutathione homoeostasis and stress response were described.^30–32^ This is of interest as Ru(III) compounds (such as KP1339) are known for their interaction with cellular glutathione pools and, thus, the redox homoeostasis.^3,20^ Consequently, the role of RLIP76 in the synergistic interaction of KP1339 with sorafenib is the matter of ongoing investigations.

Altogether, we demonstrate a promising synergism of the novel anticancer ruthenium compound KP1339 with the multi-kinase inhibitor sorafenib, which is not only based on inhibition of KP1339-induced G2/M arrest and inhibition of P38 activation but also on conversely enhanced intracellular accumulation of both drugs. The multifaceted characteristics of this synergism are probably also the reason why mostly synergistic or at least additive effects were observed in many cell lines independent of the original tumour type. This indicates that occurrence of intrinsic resistance against the KP1339 and sorafenib combination is unlikely and suggests the further clinical development of KP1339 in combination with sorafenib as a novel therapeutic strategy in hepatoma and other solid tumours.

## Financial supports

This work was supported by the Herzfelder Familienstiftung (to P. Heffeter), the Fellinger Krebsforschungsverein (to P. Heffeter), the Austrian Federal Ministry for Science and Research (BMWF) under the GEN-AU program (GZ BMWF-70.081/0018-II/1a/2008) (to W. Berger) and the Austrian Science Fond Grant L212 (to W. Berger) and L473 (to G. Koellensperger).

## Conflict of interest statement

None declared.

## Figures and Tables

**Table 1:** Anticancer activity of sorafenib and KP1339 monotreatment.

Tissue	Cell line	Sorafenib (μM)		KP1339 (μM)	
		Mean	SD	Mean	SD
Hepatoma	Hep3B	>10	–	186.3	±6.0
	HepG2	4.9	±1.6	165.4	±11.5
	PLC/PRF/5	7.2	±1.3	124.4	±2.8
	HCC1.1	>10	–	>200	–
	HCC2	2.7	±0.3	69.4	±5.8

Melanoma	VM-1	9.5	±0.2	178.3	±10.2
	VM-21	5.7	±0.9	111.7	±10.9
	VM-48	9.7	±0.2	143.8	±12.4

Lung cancer	A549	4.2	±0.2	126.4	±5.8
	VL-8	>10	–	106.1	±3.6

Colon cancer	SW480	>10	–	74.3	±1.3
	HCT116	3.4	±0.2	44.4	±2.6

## Appendix A Supplementary data

Supplementary data 1 This document contains a section on ‘materials and methods’ and Supplementary Tables 1 and 2.

Supplementary data 2 This document contains Supplementary Figs. S1–S10.
