# Supplementary material for: The ruthenium compound KP1339 potentiates the anticancer activity of sorafenib in vitro and in vivo
Source: Eur J Cancer. 2013 Oct;49(15):3366–75. doi: 10.1016/j.ejca.2013.05.018 (PMC3807657; doi:10.1016/j.ejca.2013.05.018)
Supplement: Supplementary data 1 — This document contains a section on ‘materials and methods’ and Supplementary Tables 1 and 2. [file mmc1.docx]

**Supporting Information**

**The ruthenium compound KP1339 potentiates the anticancer activity of sorafenib in vitro and in vivo**

Petra Heffeter^1,2*^, Bihter Atil^1*^, Kushtrim Kryeziu^1^, Diana Groza^1^, Gunda Koellensperger^2^, Wilfried Körner^3^, Ute Jungwirth^1^, Thomas Mohr^1^, Bernhard K. Keppler^2,4^, Walter Berger^1,2🖂^

**Table of contents**

Materials and methods

Supplementary Table 1. Details on the cell lines used in this study

Supplementary Table 2. Antibodies used in this study

References

**Total Ru uptake levels.** Cells (1x10^5^/well) were exposed to the test compounds for 3 h at 37°C. After 3 washes with ice-cold PBS, cells were lysed at room temperature in 400 µl tetramethylammonium hydroxide. Lysates were diluted in 0.6 N HNO_3_ and ruthenium concentrations determined by ICP-MS (Elan 6100; Perkin Elmer/Sciex Corporation). As unspecific binding to cell culture plastic is known for KP1019 (1), results were corrected for ruthenium (Ru) levels of a blank well containing no cells.

**LC-MS quantification of sorafenib.** The Agilent cap-LC-MSD TOF system (6210 TOF-MS, 1200 LC, Agilent Technologies, Palo Alto, California, USA) was used for determination of sorafenib in cytosoles (isolated as published (2)). The samples were diluted in acetonitrile and filtrated using Whatman Mini-UniPrep™ syringeless filters for protein removal. For chromatographic separation a reversed phase C18 column (5 µm, 20 x 4 mm, Mercury, Phenomenex, USA) was used at 40°C (injection volume 10 µl, flow rate 0.5 ml/min). The mobile phase consisted of (A) 10 mM ammonium acetate, 0.1% formic acid, pH 3.5 and (B) 99% acetonitrile, 1% water, 0.1% formic acid. The measurement uncertainty was in all cases about 10%.

**Supplementary Table 1.** Details on the cell lines used in this study

| **Cell line** | **Tissue** | **Details** | **Medium** | **Source** | **Reference** |
| --- | --- | --- | --- | --- | --- |
| Hep3B | Hepatoma | wt kras, mut P53 | RPMI 1640 | ATCC |  |
| HepG2 | Hepatoma | wt kras, wt P53 | Dulbecco's minimum essential medium | ATCC |  |
| PLC/PRF/5 | Hepatoma | mut P53, wt kras | RPMI 1640 | ATCC |  |
| HCC1.1 | Hepatoma | wt kras | RPMI 1640 | ICR | (3) |
| HCC2 | Hepatoma | wt kras | RPMI 1640 | ICR | (3) |
| VM-1 | Melanoma | mut braf (V600E) | RPMI 1640 | ICR | (4) |
| VM-21 | Melanoma | mut braf (V600E) | RPMI 1640 | ICR | (4) |
| VM-48 | Melanoma | mut braf (V600E) | RPMI 1640 | ICR | (4) |
| A549 | NSCLC | wt braf, mut kras (G12S), wt P53 | RPMI 1640 | ATCC |  |
| VL-8 | NSCLC | wt kras | RPMI 1640 | ICR | (5) |
| SW480 | Colon carcinoma | wt braf, mut kras (G12V), P53mut | minimum essential medium | ATCC |  |
| HCT116 | Colon carcinoma | P53wt, mut kras (G12S) | McCoy´s Medium | ATCC |  |

ATCC, American Type Culture Collection, Rockville, MD, USA; ICR, Institute of Cancer Research Vienna, NSCLC, non-small cell lung

**Supplementary Table 2.** Antibodies used in this study

| detected protein | species | dilution | company |
| --- | --- | --- | --- |
| PARP | rabbit | 1:1000 | Cell Signalling Technology |
| cleaved PARP | rabbit | 1:1000 | Cell Signalling Technology |
| P38 | rabbit | 1:1000 | Cell Signalling Technology |
| pP38 (Thr180/Tyr182) | rabbit | 1:1000 | Cell Signalling Technology |
| CREB | mouse | 1:1000 | Cell Signalling Technology |
| pCREB (Ser133) | rabbit | 1:1000 | Cell Signalling Technology |
| STAT3 | rabbit | 1:2000 | Cell Signalling Technology |
| pSTAT3 (Tyr 705) | rabbit | 1:1000 | Cell Signalling Technology |
| β-actin AC-15 | mouse | 1:1000 | Sigma |
| cleaved caspase 7 | rabbit | 1:1000 | Cell Signalling Technology |

PARP, anti-poly(ADP-ribosyl)polymerase;

1. Egger A, Rappel C, Jakupec MA, Hartinger CG, Heffeter P, Keppler BK. Development of an experimental protocol for uptake studies of metal compounds in adherent tumor cells. J Anal At Spectrom 2009;24:51-61.

2. Heffeter P, Bock K, Atil B, Reza Hoda MA, Korner W, Bartel C, et al. Intracellular protein binding patterns of the anticancer ruthenium drugs KP1019 and KP1339. J Biol Inorg Chem 2010;15(5):737-48.

3. Sagmeister S, Eisenbauer M, Pirker C, Mohr T, Holzmann K, Zwickl H, et al. New cellular tools reveal complex epithelial-mesenchymal interactions in hepatocarcinogenesis. Br J Cancer 2008;99(1):151-9.

4. Berger W, Hauptmann E, Elbling L, Vetterlein M, Kokoschka EM, Micksche M. Possible role of the multidrug resistance-associated protein (MRP) in chemoresistance of human melanoma cells. Int J Cancer 1997;71(1):108-15.

5. Berger W, Elbling L, Hauptmann E, Micksche M. Expression of the multidrug resistance-associated protein (MRP) and chemoresistance of human non-small-cell lung cancer cells. Int J Cancer 1997;73(1):84-93.
