# Supplementary material for: The ruthenium compound KP1339 potentiates the anticancer activity of sorafenib in vitro and in vivo
Source: Eur J Cancer. 2013 Oct;49(15):3366–75. doi: 10.1016/j.ejca.2013.05.018 (PMC3807657; doi:10.1016/j.ejca.2013.05.018)
Supplement: Supplementary data 2 — This document contains Supplementary Figs. S1–S10. [file mmc2.pptx]

## Slide 1
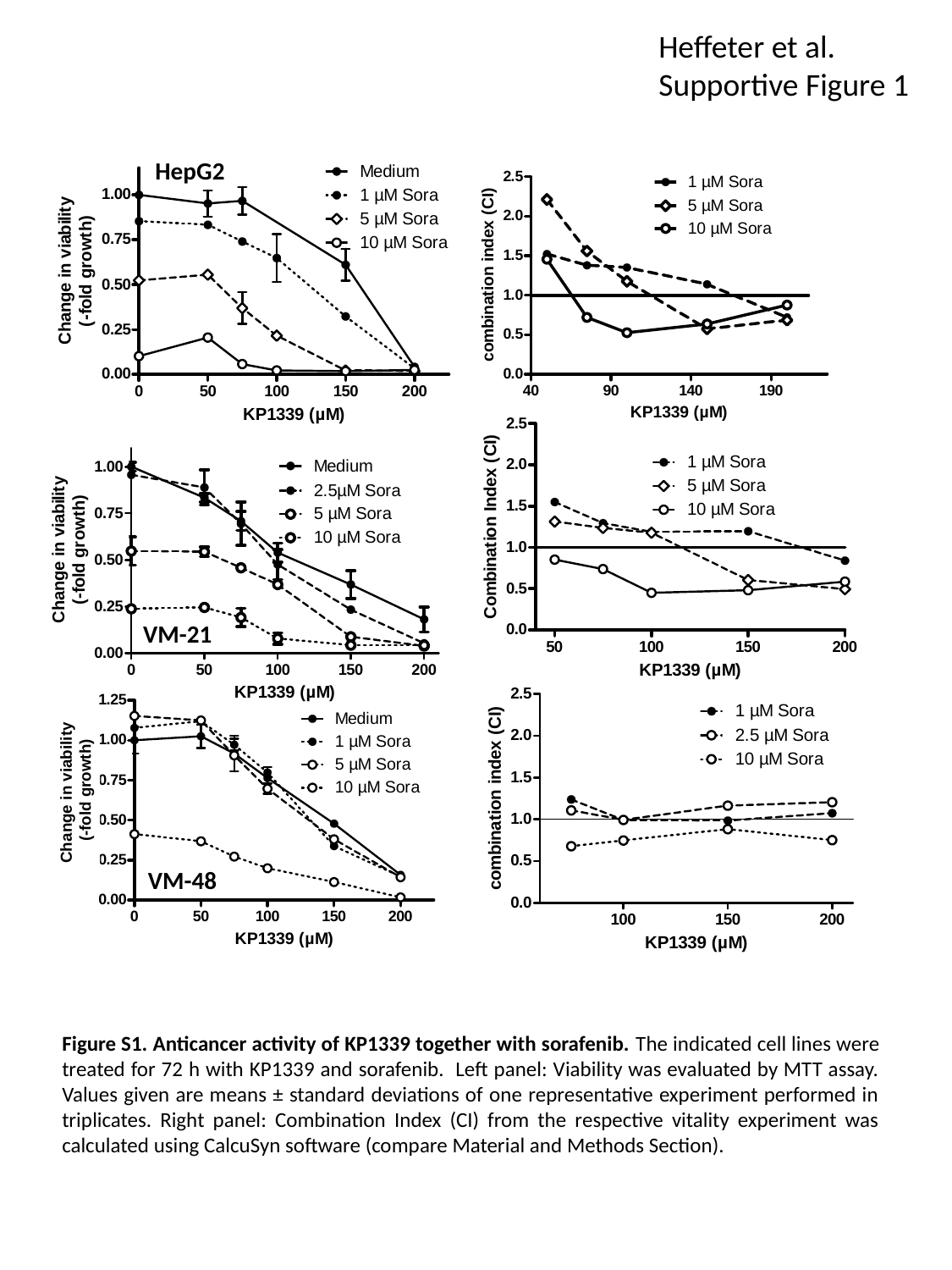

Heffeter et al.
Supportive Figure 1
HepG2
VM-21
Change in viability
(-fold growth)
VM-48
Figure S1. Anticancer activity of KP1339 together with sorafenib. The indicated cell lines were treated for 72 h with KP1339 and sorafenib. Left panel: Viability was evaluated by MTT assay. Values given are means ± standard deviations of one representative experiment performed in triplicates. Right panel: Combination Index (CI) from the respective vitality experiment was calculated using CalcuSyn software (compare Material and Methods Section).

## Slide 2
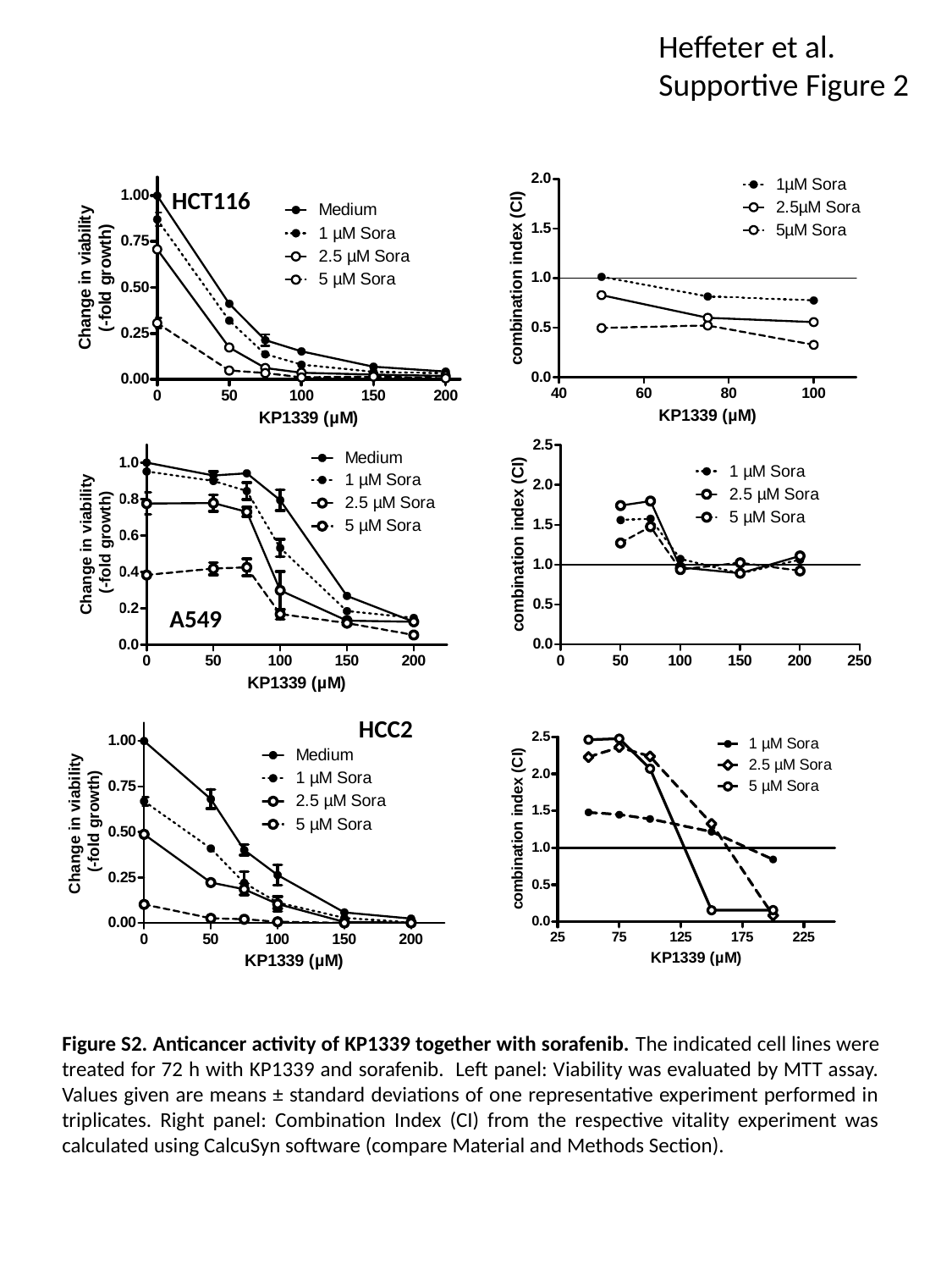

Heffeter et al.
Supportive Figure 2
HCT116
Change in viability
(-fold growth)
A549
HCC2
Change in viability
(-fold growth)
Figure S2. Anticancer activity of KP1339 together with sorafenib. The indicated cell lines were treated for 72 h with KP1339 and sorafenib. Left panel: Viability was evaluated by MTT assay. Values given are means ± standard deviations of one representative experiment performed in triplicates. Right panel: Combination Index (CI) from the respective vitality experiment was calculated using CalcuSyn software (compare Material and Methods Section).

## Slide 3
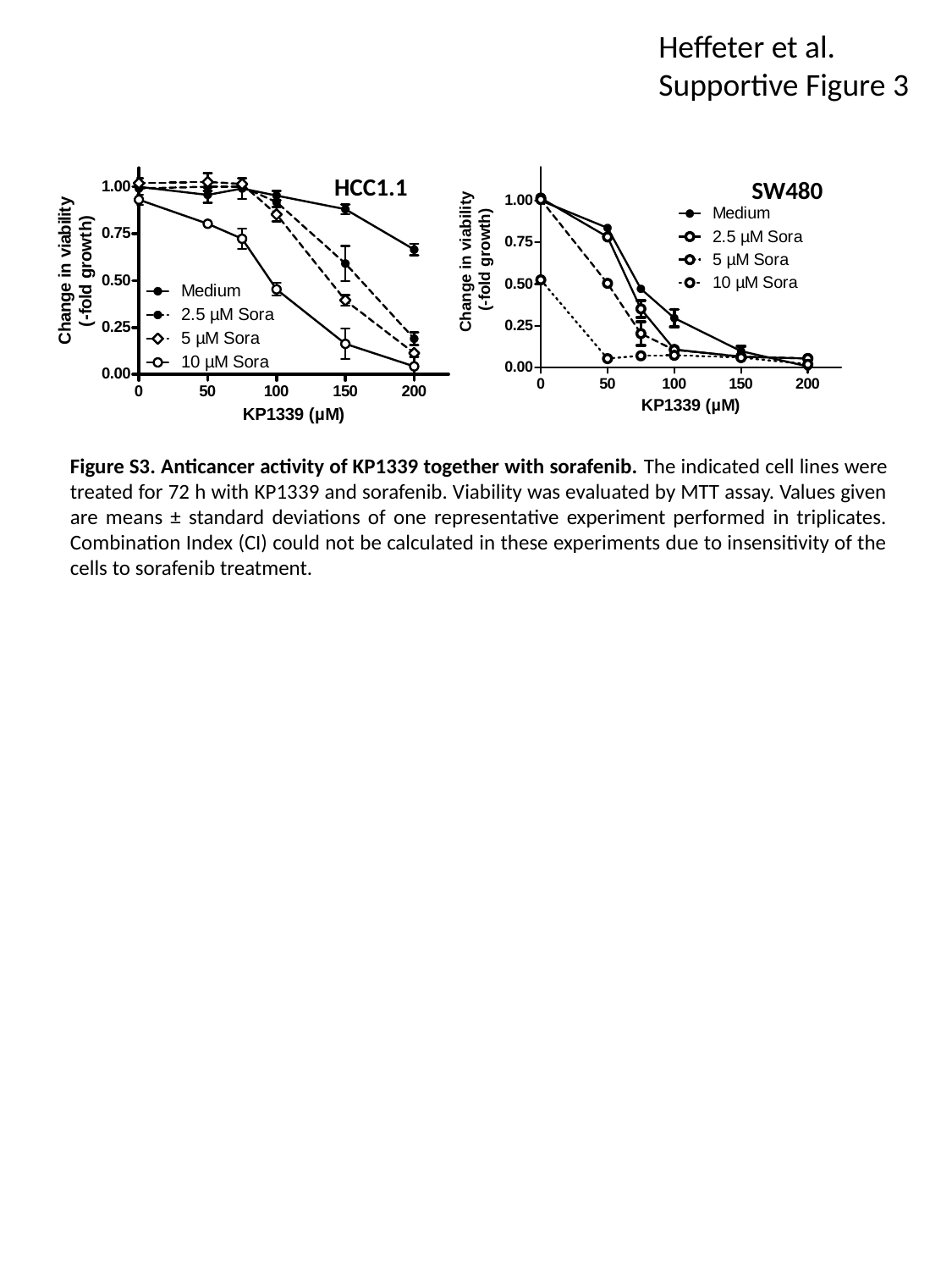

Heffeter et al.
Supportive Figure 3
HCC1.1
SW480
Change in viability
(-fold growth)
Figure S3. Anticancer activity of KP1339 together with sorafenib. The indicated cell lines were treated for 72 h with KP1339 and sorafenib. Viability was evaluated by MTT assay. Values given are means ± standard deviations of one representative experiment performed in triplicates. Combination Index (CI) could not be calculated in these experiments due to insensitivity of the cells to sorafenib treatment.

## Slide 4
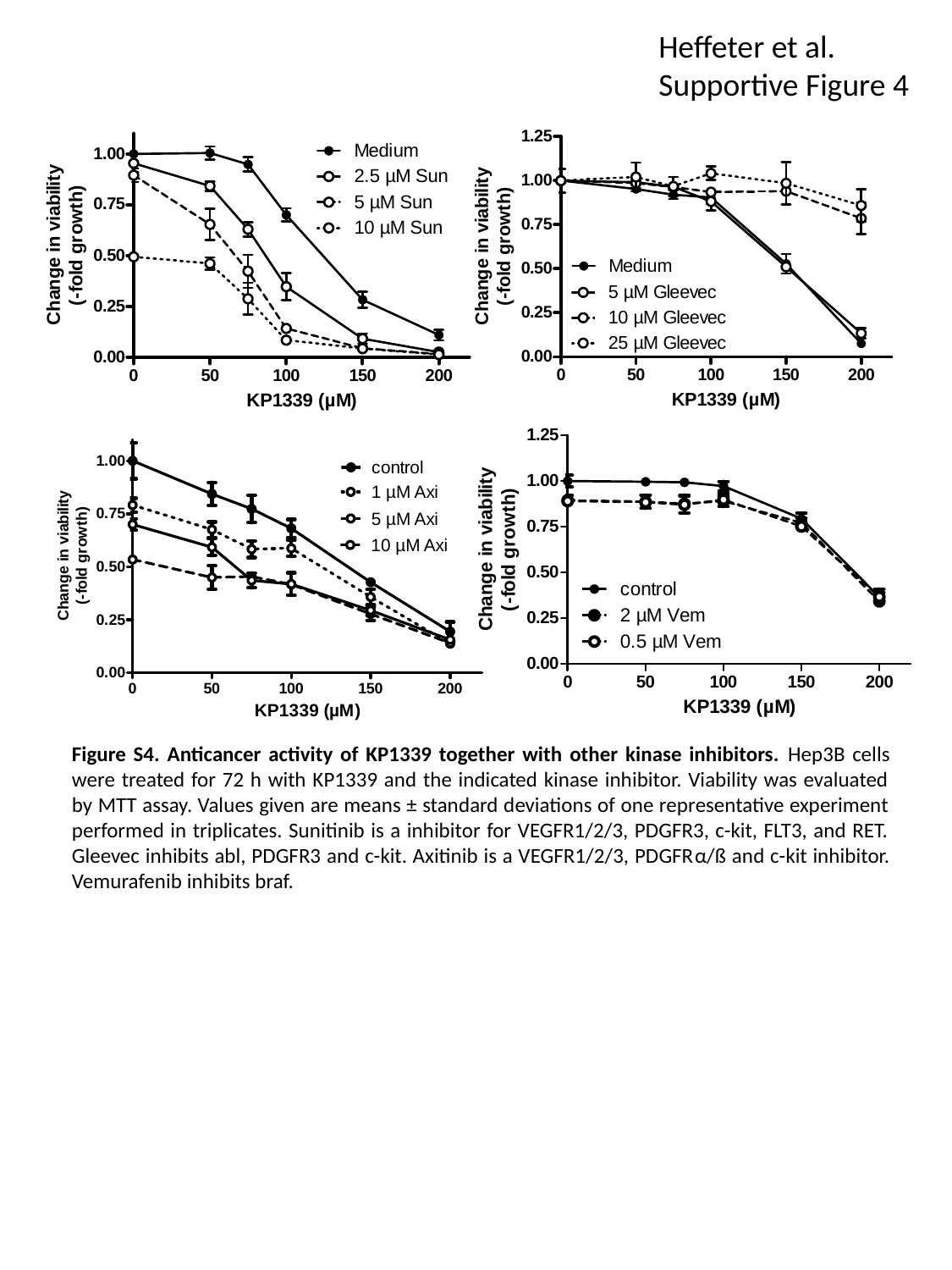

Heffeter et al.
Supportive Figure 4
Figure S4. Anticancer activity of KP1339 together with other kinase inhibitors. Hep3B cells were treated for 72 h with KP1339 and the indicated kinase inhibitor. Viability was evaluated by MTT assay. Values given are means ± standard deviations of one representative experiment performed in triplicates. Sunitinib is a inhibitor for VEGFR1/2/3, PDGFR3, c-kit, FLT3, and RET. Gleevec inhibits abl, PDGFR3 and c-kit. Axitinib is a VEGFR1/2/3, PDGFRα/ß and c-kit inhibitor. Vemurafenib inhibits braf.

## Slide 5
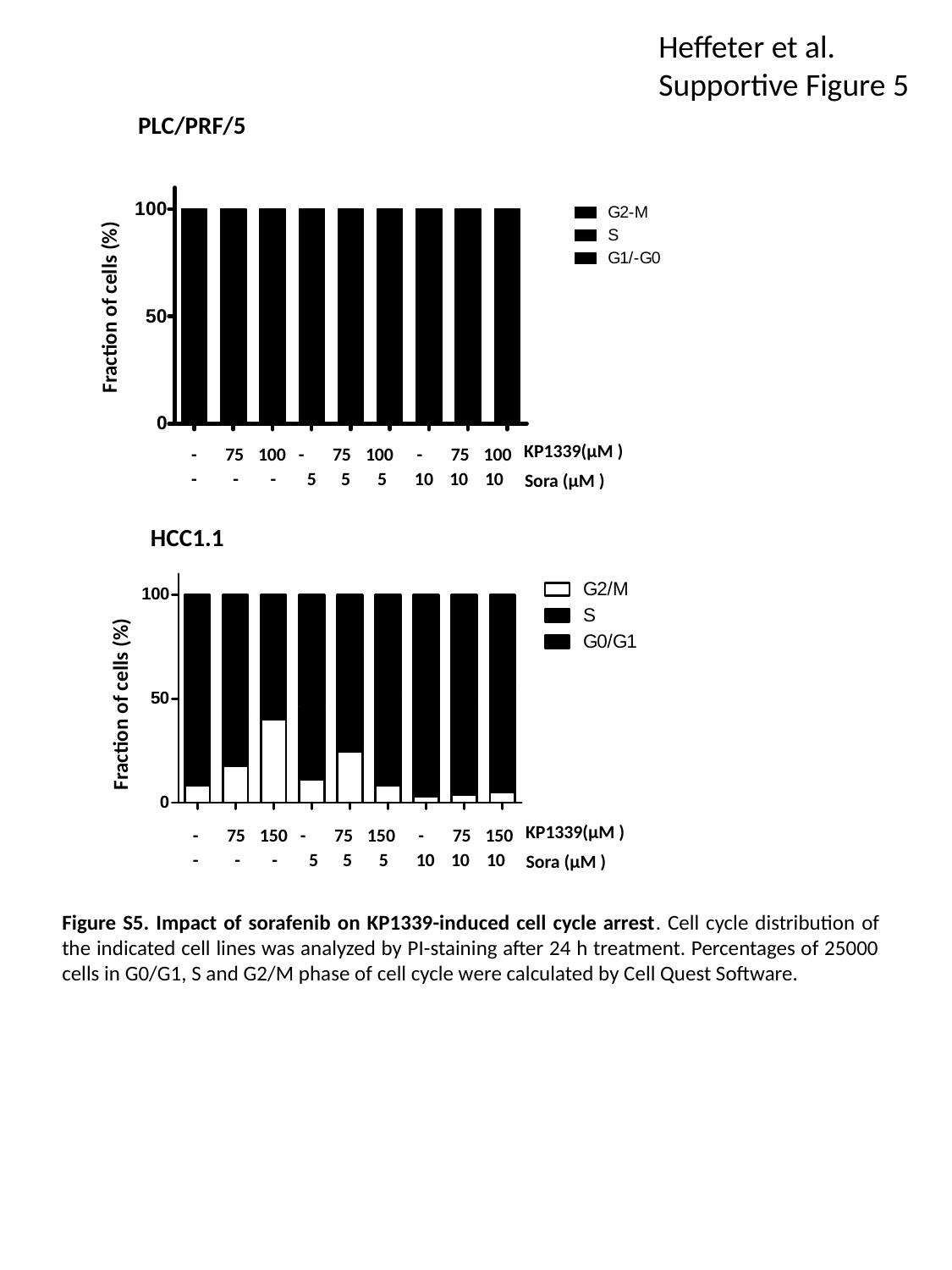

Heffeter et al.
Supportive Figure 5
PLC/PRF/5
Fraction of cells (%)
KP1339(µM )
-
75
100
-
75
100
-
75
100
-
-
5
5
5
10
10
10
-
Sora (µM )
HCC1.1
Fraction of cells (%)
KP1339(µM )
-
75
150
-
75
150
-
75
150
-
-
5
5
5
10
10
10
-
Sora (µM )
Figure S5. Impact of sorafenib on KP1339-induced cell cycle arrest. Cell cycle distribution of the indicated cell lines was analyzed by PI-staining after 24 h treatment. Percentages of 25000 cells in G0/G1, S and G2/M phase of cell cycle were calculated by Cell Quest Software.

## Slide 6
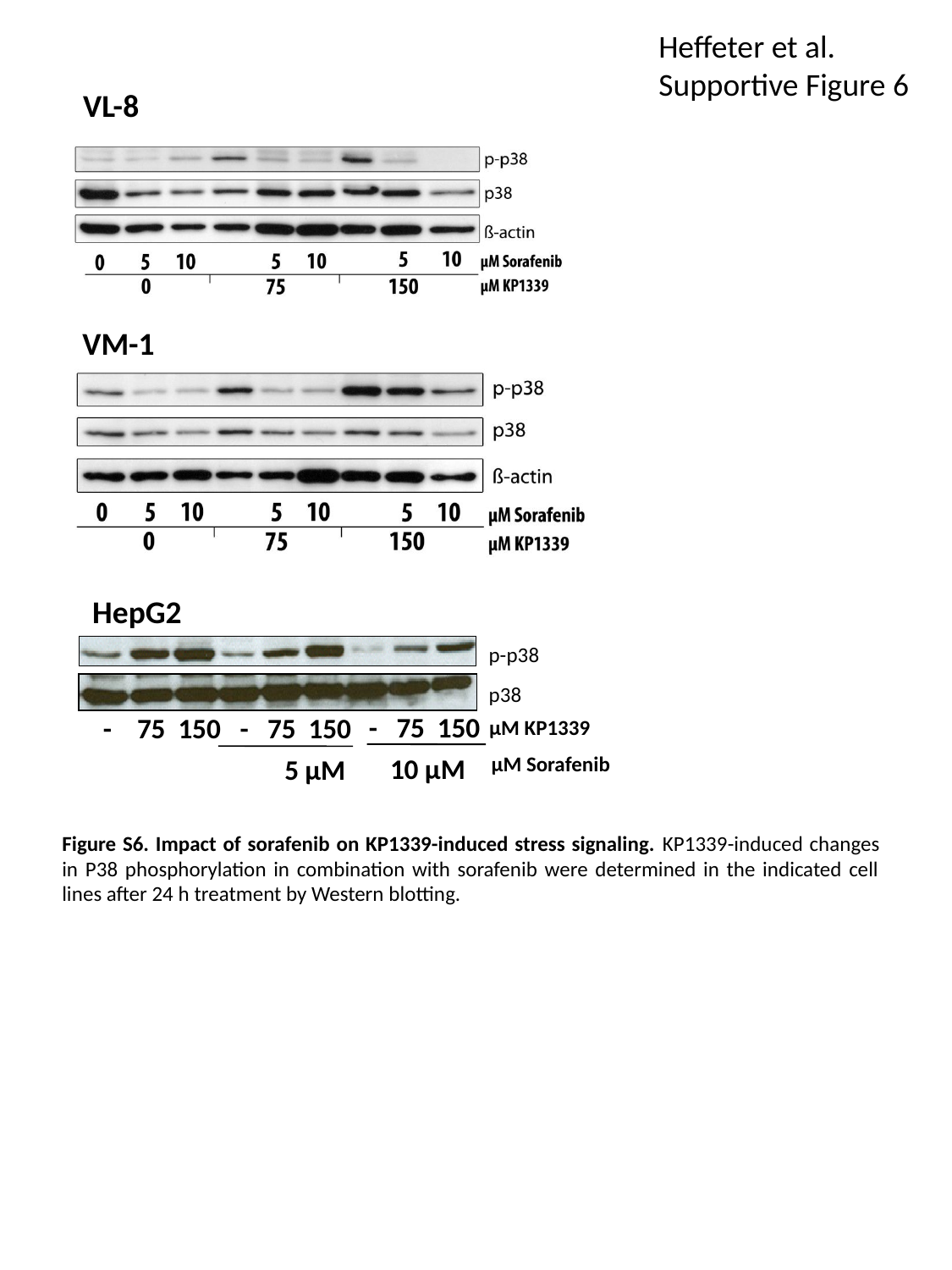

Heffeter et al.
Supportive Figure 6
VL-8
VM-1
HepG2
p-p38
p38
- 75 150
- 75 150
- 75 150
µM KP1339
µM Sorafenib
10 µM
 5 µM
Figure S6. Impact of sorafenib on KP1339-induced stress signaling. KP1339-induced changes in P38 phosphorylation in combination with sorafenib were determined in the indicated cell lines after 24 h treatment by Western blotting.

## Slide 7
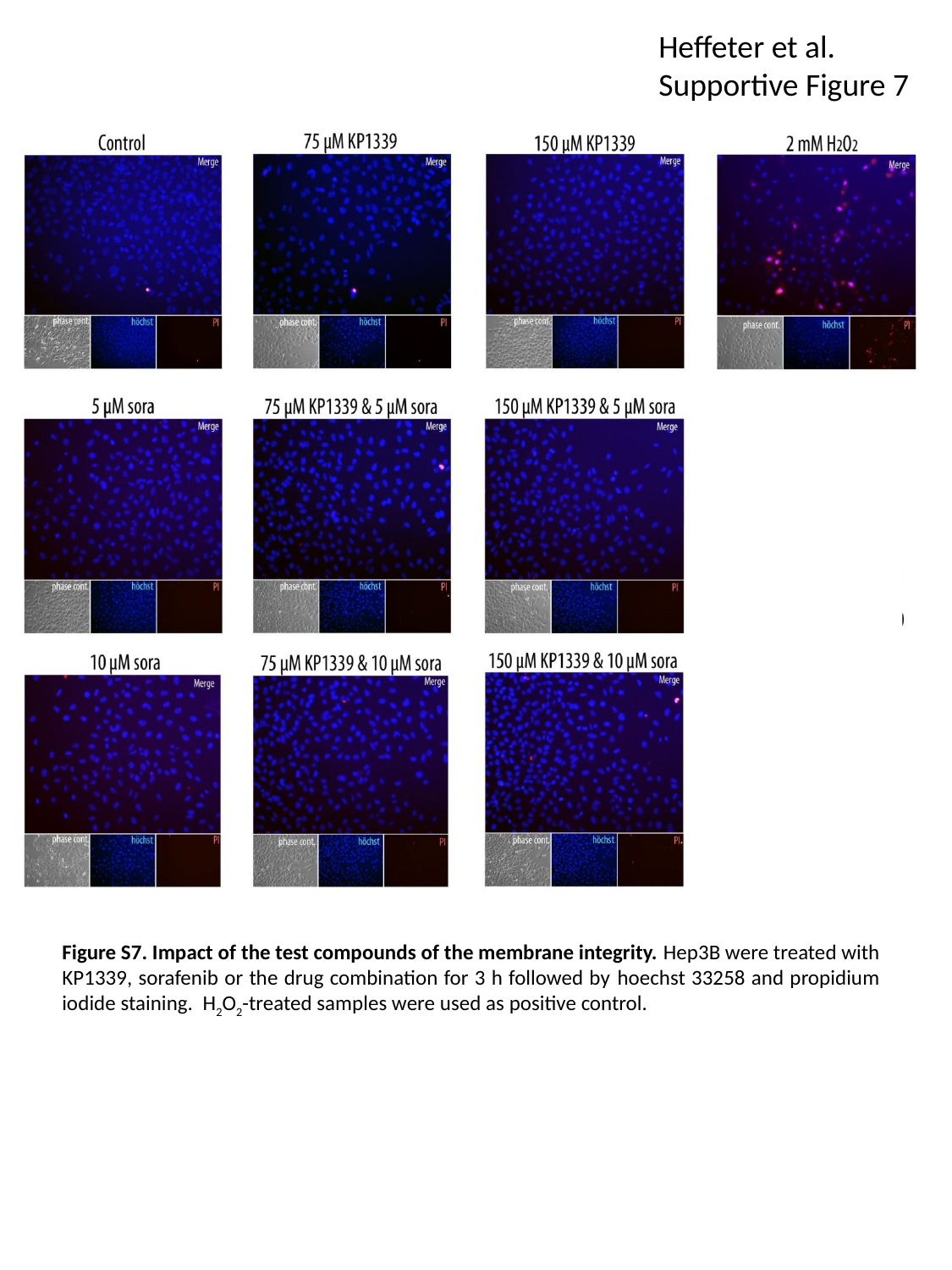

Heffeter et al.
Supportive Figure 7
Figure S7. Impact of the test compounds of the membrane integrity. Hep3B were treated with KP1339, sorafenib or the drug combination for 3 h followed by hoechst 33258 and propidium iodide staining. H2O2-treated samples were used as positive control.

## Slide 8
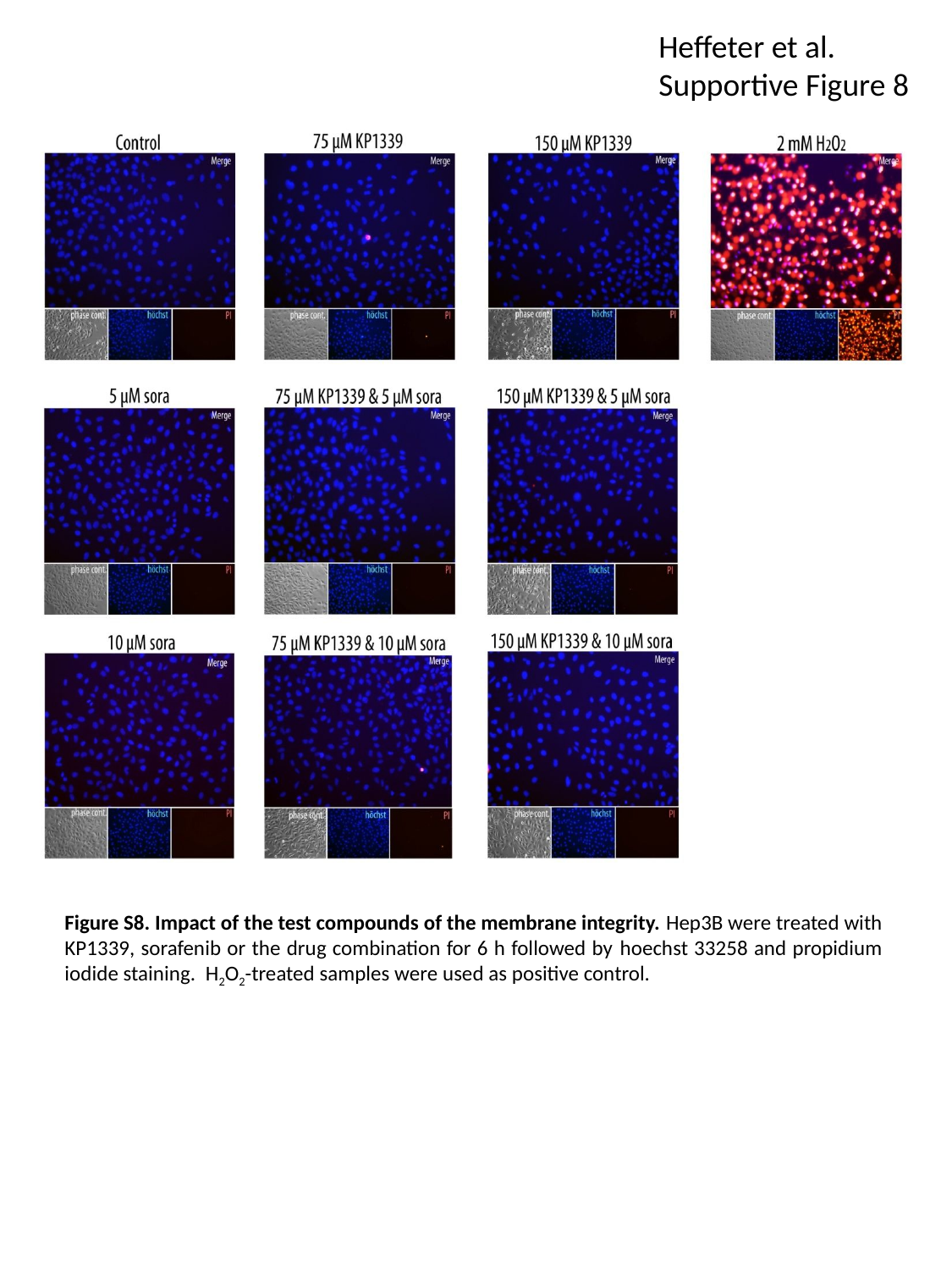

Heffeter et al.
Supportive Figure 8
Figure S8. Impact of the test compounds of the membrane integrity. Hep3B were treated with KP1339, sorafenib or the drug combination for 6 h followed by hoechst 33258 and propidium iodide staining. H2O2-treated samples were used as positive control.

## Slide 9
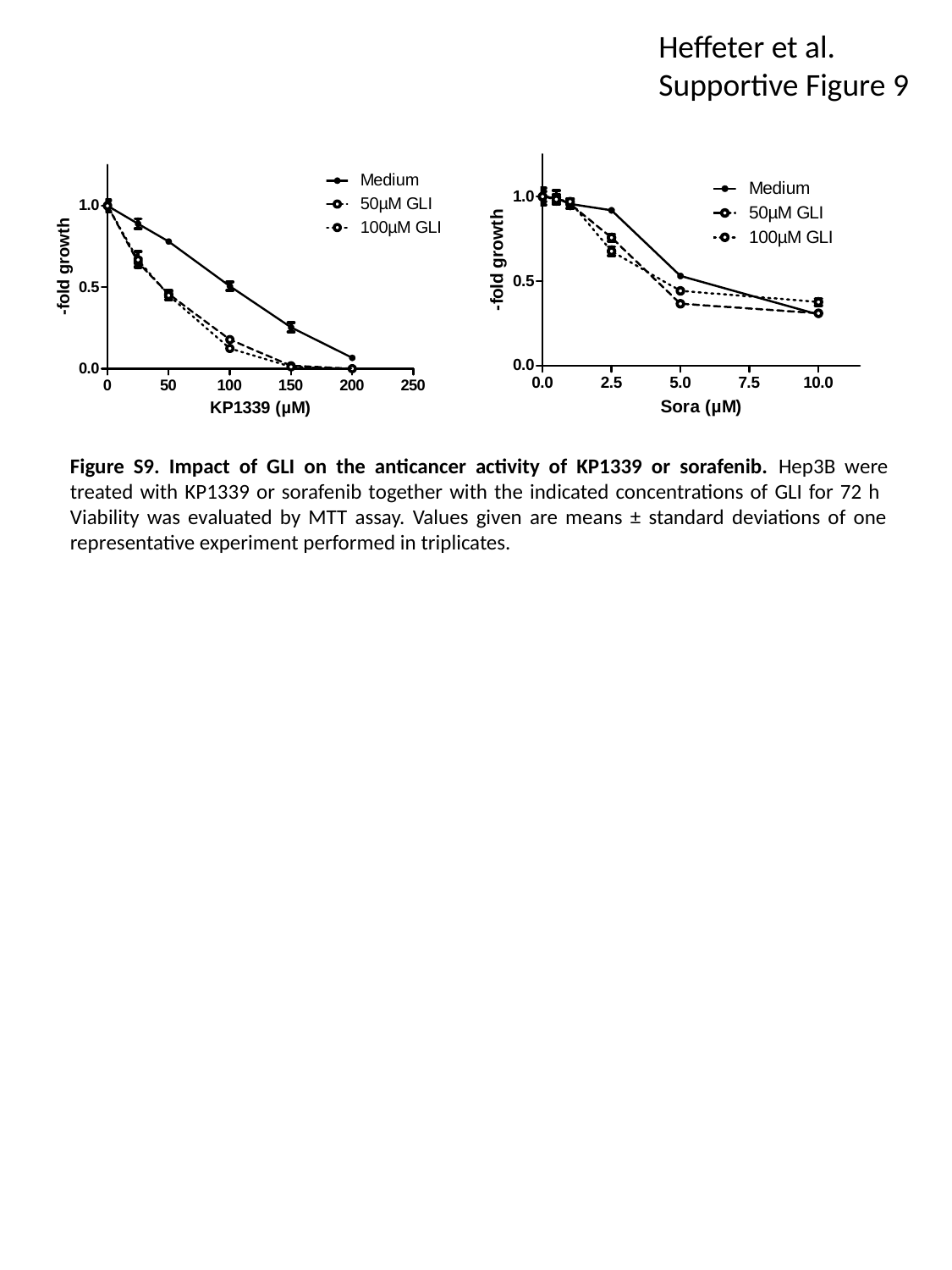

Heffeter et al.
Supportive Figure 9
Figure S9. Impact of GLI on the anticancer activity of KP1339 or sorafenib. Hep3B were treated with KP1339 or sorafenib together with the indicated concentrations of GLI for 72 h Viability was evaluated by MTT assay. Values given are means ± standard deviations of one representative experiment performed in triplicates.

## Slide 10
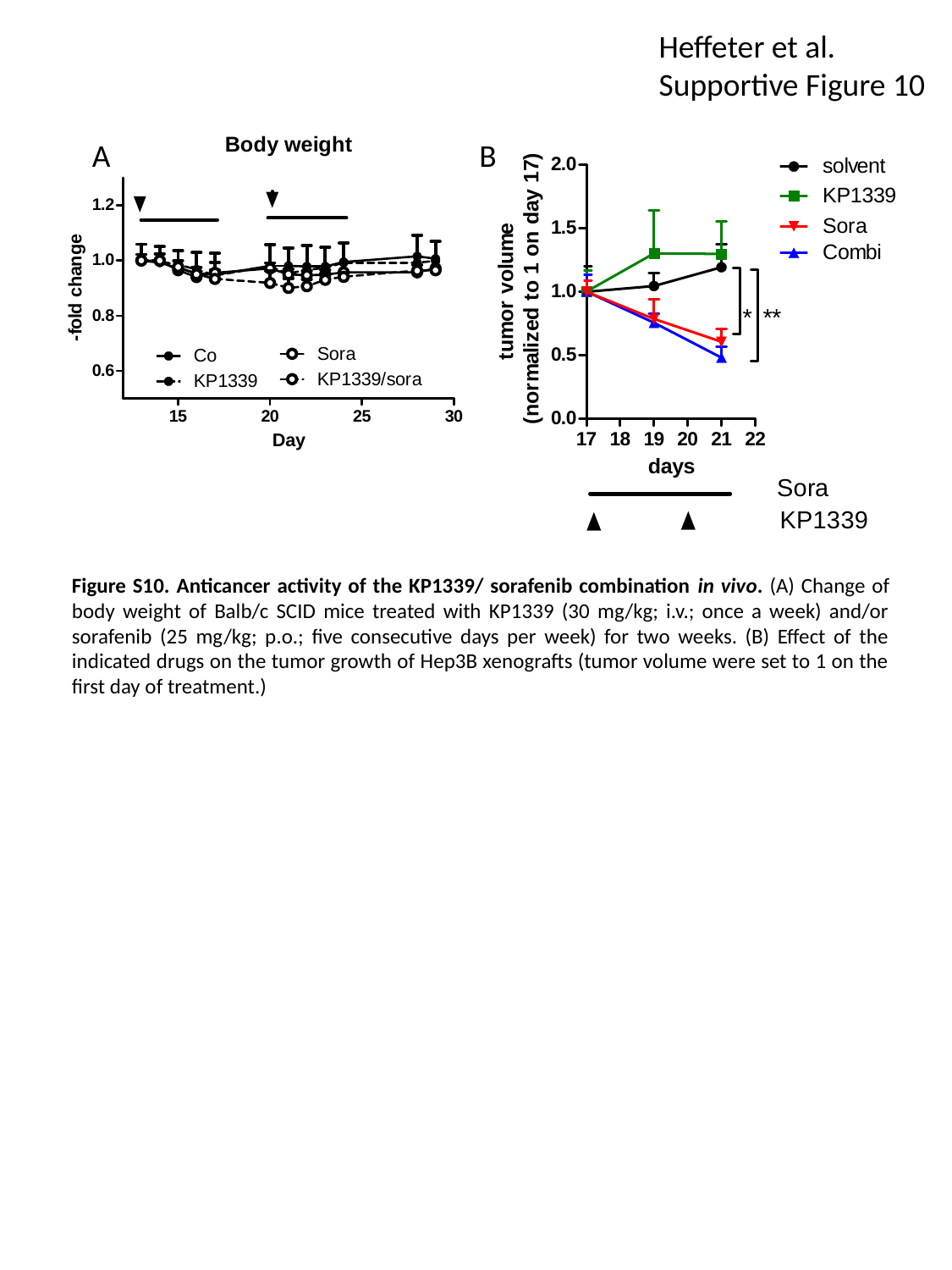

Heffeter et al.
Supportive Figure 10
A
B
Figure S10. Anticancer activity of the KP1339/ sorafenib combination in vivo. (A) Change of body weight of Balb/c SCID mice treated with KP1339 (30 mg/kg; i.v.; once a week) and/or sorafenib (25 mg/kg; p.o.; five consecutive days per week) for two weeks. (B) Effect of the indicated drugs on the tumor growth of Hep3B xenografts (tumor volume were set to 1 on the first day of treatment.)
